# Editorial: Profiling the tumour microenvironment to unveil biomarkers and develop novel therapeutics for cancer therapy

**DOI:** 10.3389/fmed.2023.1178532

**Published:** 2023-03-22

**Authors:** Sebastien Jaillon, Diletta Di Mitri

**Affiliations:** ^1^Department of Biomedical Sciences, Humanitas University, Milan, Italy; ^2^IRCCS Humanitas Research Hospital, Milan, Italy

**Keywords:** cancer, tumor microenvironment, prognostic markers, NKT, cell death

A link between inflammation and cancer has been proposed in the 19th century and the concept of cancer-related inflammation, including the presence of inflammatory leukocytes in the tumor microenvironment (TME), has been established as a hallmark of cancer (1–3). The presence, phenotype and activation states of tumor-infiltrating leukocytes are controlled by soluble molecules, including cytokines and chemokines found in the TME (4). These soluble molecules are produced by different cell types, including cancer cells, immune cells, fibroblasts and endothelial cells. Therefore, the immune and inflammatory landscape of the TME is an essential component of cancer and its exploration is important for the development of new therapeutic approaches, as demonstrated with the immune checkpoint blockades (5). In recent years, the advent of molecular technologies has enabled the access to genomic and transcriptional information from cancer cells and the tumor immune microenvironment. These molecular features can be exploited to correlate the genetic background of cancer cells to the composition of its microenvironment, to predict cell-cell interactions and to derive gene signatures indicative of disease. In this context, the scope of the Research Topic “*Profiling the tumour microenvironment to unveil biomarkers and develop novel therapeutics for cancer therapy*” was to provide an overview of immune-related cellular and molecular features that can be exploited to forecast cancer prognosis and predict response to therapy.

The topic opens with a revision of current literature on the impact of invariant Natural Killer T (iNKT) cells in cancers and proceeds with issues that discuss the implications of specific gene scores in tumor progression and their potential correlation with the composition of the TME.

Unconventional T cells represent a heterogeneous group of T cells mostly characterized by a limited diversity and the recognition of a variety of non-polymorphic ligands. They include iNKT cells, which express a semi-invariant αβ T-cell receptor that recognizes lipid antigens presented in the context of the major histocompatibility complex class I-related molecule CD1d. In this Research Topic, Delfanti et al. reviewed the role played by iNKT cells in anti-tumor immune response and their potential involvement in anti-cancer therapy. In particular, iNKT cells have the capacity to kill tumor cells and to drive the reprogramming of immunosuppressive cells. This review presents the different therapeutic approaches using iNKT cells, including adoptive transfer and cells engineered to express a second T-cell receptor or a chimeric antigen receptor (Delfanti et al.).

The identification of effective cancer biomarkers useful for diagnosis, prognosis and prediction of therapy efficacy represents a major challenge. In this context, the application of genomics paved the way for approaches that stratify patients on the basis of molecular features in addition to histological characteristics. Importantly, the integration of molecular signatures with the profiling of the tumor immune infiltrate may further improve the estimation of disease progression and the prediction of response to therapy.

In this Research Topic, Li Y. et al. identified a transcriptomic signature based on the expression of Interferon Gamma Receptor 1 (IFNGR1) and IFNGR2 in gliomas that is associated with patient outcomes. Using 1,693 glioma samples retrieved from *The Cancer Genome Atlas* and *Chinese Glioma Genome Atlas*, the authors found that the expression of these molecules was higher in tumors with the highest clinical grade. Accordingly, high expression of IFNGRs was associated with poor prognosis and shorter progression-free interval. This finding supports the double-edged immunomodulatory role of IFN-γ in cancer. Indeed, although IFN-γ is known to support a type 1 immune response against tumors, this cytokine can also induce immune evasion through different mechanisms, including the expression of the IFNγ-induced molecules indoleamine 2,3- dioxygenase 1 and programmed cell death 1 ligand 1 by tumor cells or tumor-infiltrating leukocytes (6). In line with this hypothesis, the algorithm *Tumor Immune Dysfunction and Exclusion*, developed to predict the response to immune checkpoint blockade (7), showed that patients with higher expression of IFNGRs were more likely to respond to anti-programmed cell death 1 therapy (Li Y. et al.).

A second article of this Research Topic aimed to established new prognostic biomarkers for patients with gastric cancer. Although the incidence is declining, gastric cancer represents one of the most common cancers and the 4th leading cause of cancer death worldwide (8). Gastric cancer can be asymptomatic before reaching the most advanced stages, for which survival is poor. Therefore, identification of new biomarkers for prognosis is crucial. In the study by Wei et al., the authors used bioinformatic tools and human datasets to propose a list of 11 inflammation-related genes (*APOA1, CYP19A1, F5, HBB, IGFBP1, MATN3, MTTP, PON1, PVT1, RNASE3, and SERPINE1*), which are differentially expressed between normal and cancer samples, and can serve as prognostic biomarkers for gastric cancer. Based on the median score, patients have been divided into low-risk and high-risk groups. The low-risk group was associated with better prognosis and a computational approach estimating the relative proportions of the various cell subsets revealed an enrichment of CD8 T cells. The high-risk group was associated with poor prognosis and an enrichment of macrophages. Therefore, this finding paves the way for further investigations required to validate the significance of this signature and the roles of these molecules in the TME of gastric cancer.

The Research Topic also includes two reports that evaluated the prognostic value of cell death signatures in cancer patients. In recent years, the induction of non-apoptotic regulated cell death, including necroptosis, ferroptosis and pyroptosis, has been considered as a novel therapeutic approach in cancer. Non-apoptotic regulated cell death has been correlated with anti-tumor immunity and the possibility to exploit it to improve immunotherapy efficacy is fascinating. In the present issue, He et al. investigated the expression of necroptosis-related genes in colon cancer patients and identified a gene score that correlates with tumor progression and modulations of the TME. The authors identified mutations associated with necroptosis in colon cancer datasets and identified tumor molecular subtypes with diverse survival advantages and distinct TME characteristics. Additionally, the authors analyzed the expression of necroptosis-related genes in a meta-cohort composed by 1,172 colon cancer patients and derived a necroptosis risk score signature, composed by 8 genes (GPRASP1, LAMP5, GRP, FABP4, MMP10, MMP1, SPINK1, and CLCA4), that predicts progression and susceptibility to chemotherapy agents. The score correlates with distinct immune features and with the expression of immune checkpoint genes and may thus be exploited as a biomarker of immune checkpoint blockade response. On the same line, Li H. et al. constructed a prognostic index composed by 5 pyroptosis-related mRNA that was applied to predict prognosis in lung adenocarcinoma patients. A high value of this index correlates with worse overall survival independently from tumor stage and is associated with lower immune reactivity, as suggested by the evaluation of enrichment scores of immune cells and immune-related pathways by *Gene Set Enrichment Analysis*. In contrast, patients with lower values of this index showed a higher Immunephenoscore, indicative of a better response to immune checkpoint inhibitors.

Collectively, the manuscripts of this Research Topic support the importance of the TME on tumor growth and its role in prognosis and prediction of response to treatment, particularly in response to immune checkpoint inhibitors. A better understanding of the different molecular and cellular signatures of the TME is essential for the development of new cancer therapies able to target both the tumor cells and the surrounding microenvironment.

## Author contributions

All authors listed have made a substantial, direct, and intellectual contribution to the work and approved it for publication.
